# Chronic Fluoride Exposure and the Risk of Autism Spectrum Disorder

**DOI:** 10.3390/ijerph16183431

**Published:** 2019-09-16

**Authors:** Anna Strunecka, Otakar Strunecky

**Affiliations:** The Institute of Technology and Business, Okružní 517/10, 370 01 České Budějovice, Czech Republic; otakar.strunecky@gmail.com

**Keywords:** autism spectrum disorder, ASD prevalence, aluminum, endemic fluorosis, chronic fluoride exposure, immunoexcitotoxicity, neurotoxicity, socioeconomic status, water fluoridation

## Abstract

The continuous rise of autism spectrum disorder (ASD) prevalent in the past few decades is causing an increase in public health and socioeconomic concern. A consensus suggests the involvement of both genetic and environmental factors in the ASD etiopathogenesis. Fluoride (F) is rarely recognized among the environmental risk factors of ASD, since the neurotoxic effects of F are not generally accepted. Our review aims to provide evidence of F neurotoxicity. We assess the risk of chronic F exposure in the ASD etiopathology and investigate the role of metabolic and mitochondrial dysfunction, oxidative stress and inflammation, immunoexcitotoxicity, and decreased melatonin levels. These symptoms have been observed both after chronic F exposure as well as in ASD. Moreover, we show that F in synergistic interactions with aluminum’s free metal cation (Al^3+^) can reinforce the pathological symptoms of ASD. This reinforcement takes place at concentrations several times lower than when acting alone. A high ASD prevalence has been reported from countries with water fluoridation as well as from endemic fluorosis areas. We suggest focusing the ASD prevention on the reduction of the F and Al^3+^ burdens from daily life.

## 1. Introduction

Autism spectrum disorder (ASD) covers a range of heterogeneous neurodevelopmental conditions, characterized by persistent deficits in social and communication interactions and presenting with repetitive, stereotypic interests and behaviors. ASD incorporates autistic disorder, Asperger’s disorder, childhood disintegrative disorder, and pervasive developmental disorder not otherwise specified [1,2,3,4]. The word autism is traditionally used interchangeably with ASD. ASD begins early in childhood and tends to persist into adolescence and adulthood.

ASD is the fastest-growing developmental disability in the last two decades in all countries over the world. While in the period 1960–1989 autism was a rare disease with one to four per 10,000 in both Europe and America, the WHO estimates that 62 per 10,000 children (one in 160) in the world have ASD currently [2]. The rise in the ASD rate has sparked fears of an ASD epidemic, mainly in the United States (US). In US children aged 3–17 years, the estimated prevalence of ASD diagnosis, based on parental reports, is one in 40 [5]. 

Understanding of the ASD etiopathogenesis is therefore urgently warranted to find an effective prevention. A consensus suggests that the ASD etiopathogenesis is presumably multifactorial and results from very complex interactions between genetic and environmental factors [6]. The high ASD prevalence has been reported from countries with artificial water fluoridation as well as from countries with endemic fluorosis areas [7]. On the contrary, a lower ASD prevalence has been reported from some member states of the European Union (EU) [8,9,10], where water fluoridation was banned in the 1970s–1990s. Therefore, in our previous papers, we suggested that fluoride (F) might be among the key environmental factors in the ASD etiopathogenesis [7,11]. F is not currently recognized as a causative culprit of ASD, since its neurotoxic effects are not generally accepted. We assessed the risks of chronic F exposure in the ASD etiopathology regarding the F-induced effects on metabolism and mitochondrial dysfunction, oxidative stress and inflammation, immunoexcitotoxicity, and melatonin levels. Such effects have all been observed in people with ASD. Our review aims to provide evidence of F neurotoxicity. Moreover, F in the synergistic action with aluminum’s free cation (Al^3+^) in molecules of aluminofluoride complexes can trigger various metabolic and pathological symptoms. This triggering takes place at F concentrations several times lower than F acting alone [12,13,14].

Fluoridation of drinking water in addition to the extensive use of F in medicine and industry, started the era of supplementation of the living environment with these ions as never before in the history. It must be pointed out that F does not have any known essential function in human physiology and development. No signs of F deficiency have been identified [15]. 

## 2. Fluoride-Induced ASD Symptoms

F has long been known to influence the activity of various enzymes in vitro [14]. We present evidence that F induces oxidative stress, inflammation, and immunoexcitotoxicity. There is a link between a known effect of F on melatonin synthesis in the pineal gland [16] and significantly reduced melatonin synthesis in ASD [17]. 

### 2.1. Enzymes and Mitochondrial Disorders

F alters, in micromolar and millimolar concentrations, in vitro activities of many important enzymes. We reviewed that F has been found to inhibit 22 various enzymes, such as enolase, pyruvate kinase, lactate dehydrogenase, Na^+^, and K^+^-ATPase [14]. On the other hand, 20 enzymes are stimulated by F, including adenylyl cyclase, alanine transaminase, lactate dehydrogenase, and glycogen phosphorylase. It is therefore evident that F alters the energy metabolism and might trigger numerous metabolic disorders. 

It might seem that F in whole organisms may not reach the concentrations that were used in the laboratory experiments in vitro. Some studies found that under certain circumstances, the inhibitory or stimulatory impact of F can be more pronounced at a lower level of intake than at a higher level (paradoxical dose–response effects) [18]. It is therefore difficult to predict the actual effective concentrations of F in vivo.

**Figure 1:**
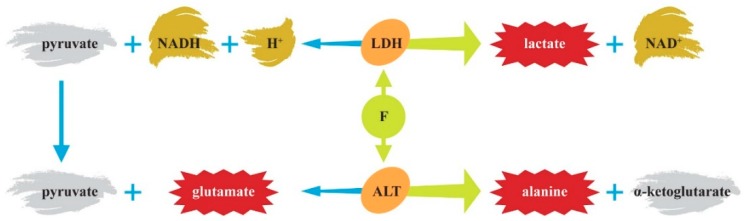
Patients with autism spectrum disorder (ASD) have increased levels of blood lactate, alanine, and glutamate. LDH—lactate dehydrogenase; ALT—alanine transaminase.

However, autistic patients display peripheral markers of mitochondrial energy metabolism dysfunctions, such as elevated lactate and alanine levels in the blood (Figure 1) [19]. Even though alterations in the mitochondrial and cellular energy metabolism are not specific for ASD, they indicate the potential pathological events that might be induced by F. 

Evidence of mitochondrial dysfunction was reported among 73 Egyptian children with autism who were compared with another 73 healthy age-matched children. Serum lactate, pyruvate, creatine kinase, L-carnitine, lactate dehydrogenase, pyruvate kinase, alanine transaminase, and aspartate transaminase were measured [20]. The plasma levels of lactate and the lactate/pyruvate ratio were significantly higher among autistic children than in the control group.

Mitochondrial dysfunction and its link to ASD symptoms were investigated [21]. The authors found that overall, 62% of 76 ASD individuals showed mitochondrial enzyme activity outside the control rates of individuals without significant chronic health conditions. This study demonstrated for the first time that such variations are related to the social function and behavior of ASD persons. Clinical aspects of mitochondrial dysfunction in ASD might include unusual neurodevelopmental regression, especially if triggered by an inflammatory event, gastrointestinal symptoms, seizures, motor delays, fatigue, and lethargy [22,23]. 

### 2.2. A Synergy of F and Al^3+^ in the ASD Etiopathology

Fluoride reacts with trace amounts of Al^3+^ within body fluids and produces aluminofluoride complexes [12,13,14,24,25]. Aluminum tetrafluoride (AlF_4_^−^) is a molecule whose shape and physical properties closely resemble those of the phosphate anion, PO_4_^3−^. Since the 1980s, AlF_4_^−^ has been widely used in laboratory investigations as an analog of phosphate groups to study phosphoryl transfer reactions and heterotrimeric G protein involvement in signal transduction. However, an important functional difference between a phosphate group and the structurally analogous AlF_4_^−^ exists [26]. In phosphate, oxygen is covalently bound to the phosphorus and does not exchange with oxygen from a solvent, while in AlF_4_^−^, the bonding between the electropositive Al^3+^ and the highly electronegative F is more ionic, allowing F in the bound complex to exchange freely with F ions in solution. While the reaction of a bound phosphate with orthophosphate is endergonic and slow, the corresponding reaction with AlF_4_^−^ is rapid and spontaneous.

AlF_4_^−^ binds ionically to the terminal oxygen of ADP or GDP β-phosphate. ADP or GDP could, therefore, form a complex with AlF_4_^−^ that imitates ATP or GTP in their effect on protein conformation. This effect causes a structural change that locks the site and prevents the release of the γ-phosphate. The interactions of AlF_4_^−^ with signaling cascades of G protein-coupled receptors (GPCRs) have been documented by several authors using both biochemical as well as X-ray crystallographic analysis [27,28,29].

**Figure 2:**
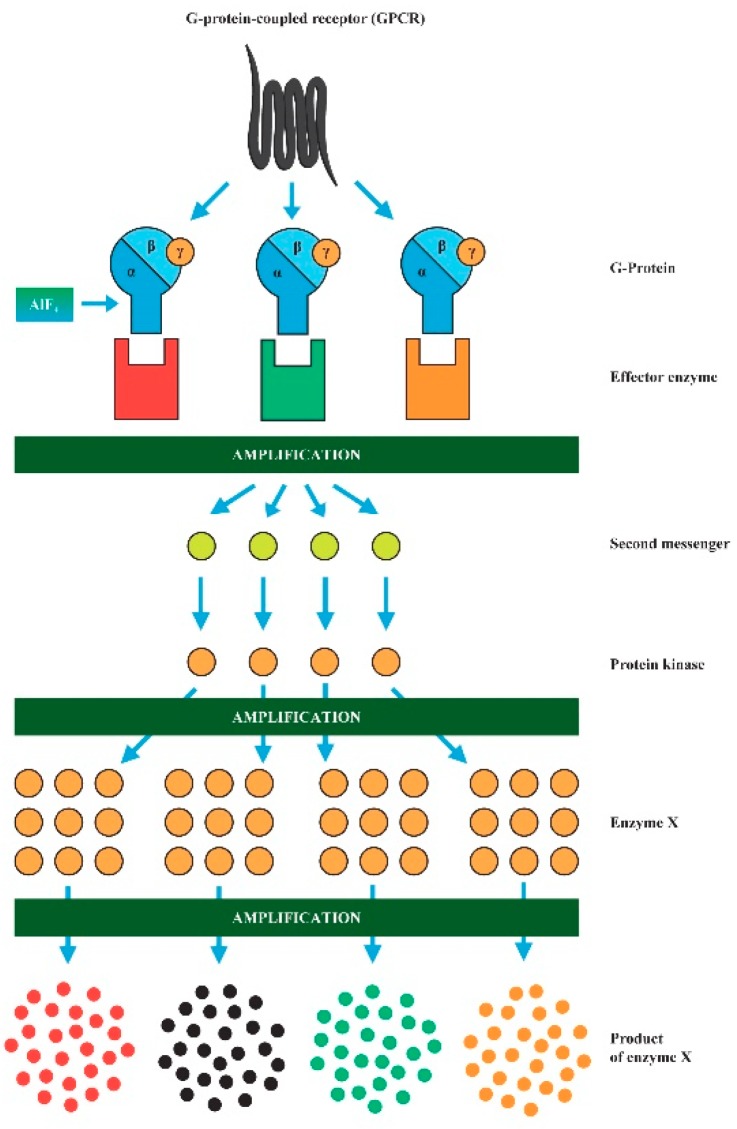
Amplification of signaling pathways by AlF_4_^−^. Its message is greatly amplified during the conversion into the functional response of a cell. Effector enzymes are adenylyl cyclase or phospholipase C. The second messenger molecule could be cAMP, inositol 1,4,5- trisphosphate, and diacylglycerol.

Physiological agonists of GPCRs include neurotransmitters and hormones, such as glutamate, dopamine, serotonin, melatonin, acetylcholine, and neuropeptides [30]. AlF_4_^−^ is a molecule sending a false message. The false signal of AlF_4_^−^ is amplified during its conversion into a functional response (Figure 2). Even very low F concentration in synergy with Al^3+^ can exacerbate alterations in neurotransmission and hormonal regulation. AlF_4_^−^ can thus evoke a whole network of pathological events in micromolar concentrations. It has the potential to modulate neurodevelopment, brain structure, structural plasticity, as well as higher neuronal functions. The detailed description of molecular targets of AlF_4_^−^ relevant to the ASD pathogenesis was discussed in our previous review [11]. Recently, Al^3+^ has been regarded as nontoxic, and it is a component of pot water, food, beverages, medicine, and cosmetics.

### 2.3. The Effects of F in Oxidative Stress, Inflammation, and Immunoexcitotoxicity

**Figure 3:**
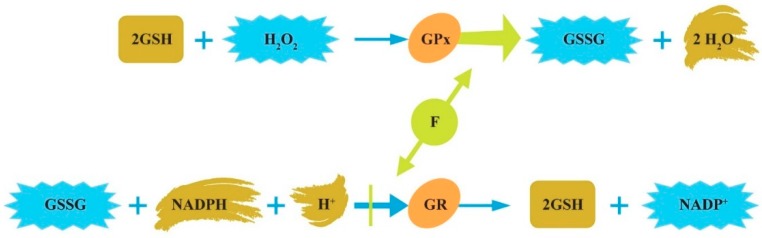
The glutathione redox system. Glutathione (GSH), a radical scavenger, is converted to oxidized glutathione (GSSG) through GSH peroxidase (GPx) and converted back to GSH by GSH reductase (GR). GSH can detoxify hydrogen peroxide (H_2_O_2_), preventing the formation of free radical generation and lipid peroxidation products.

Oxidative stress, defined as an imbalance between oxidants and antioxidants in favor of the oxidants, represents the link between genetic, epigenetic, immunological, and environmental factors underlying ASD. The glutathione (GSH) redox system is most important for reducing oxidative stress. A decrease in GSH is one of the best-documented biochemical changes in plasma, immune cells, and brains of children with ASD [11,20,31,32,33,34]. F exposure can reduce the cellular level of GSH and induce oxidative stress (Figure 3). 

The oxidative stress in the pathology of endemic fluorosis in the blood of the population living in the areas with severe coal-burning endemic fluorosis in China was also reported [7,35]. The activities of superoxide dismutase, GSH reductase (GR), and catalase were significantly decreased in blood plasma and erythrocytes of children and adults with a low level of GSH. 

**Figure 4:**
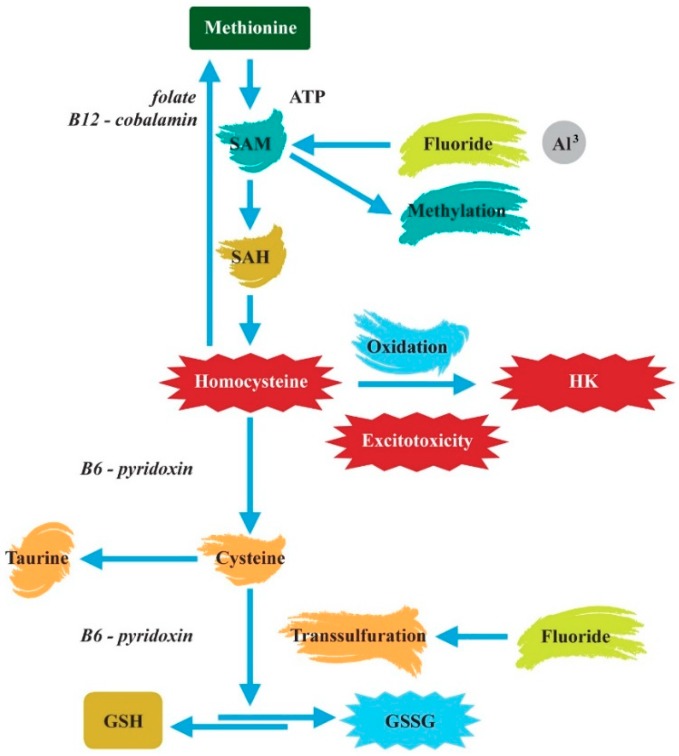
Methylation and transsulfuration metabolism in ASD. SAM—S-adenosyl methionine, SAH—S-adenosylhomocysteine, GSH—glutathione, GSSG—oxidized glutathione, ATP—adenosine triphosphate.

GSH synthesis and intracellular redox balance are linked to folate and methylation metabolism, metabolic pathways that have also been shown to be abnormal in ASD [33,36] (Figure 4). DNA methylation changes in autistic cerebral cortex regions were described [37]. Additionally, along with F, Al^3^^+^ disrupts enzymes involved in the methylation pathways [11,14]. 

Reduced levels of GSH greatly increase the sensitivity of neurons and astrocytes to oxidative stress and excitotoxicity. Oxidative stress induces the secretion of many vasoactive and proinflammatory molecules, which leads to neuroinflammation. The initial reaction to inflammation may be the activation of microglia, the resident immune cells in the brain [11,38]. Several groups of researchers and clinicians have reported inflammation in the brain of both young and old individuals with ASD. Vargas et al. [39] demonstrated an active neuroinflammatory process in brain tissues and the cerebrospinal fluid (CSF) in autistic patients. Brain tissues obtained at autopsy from 11 patients with autism were used for morphological studies. Fresh-frozen tissues available from seven patients and CSF from six living autistic patients were used for cytokine protein profiling. Immunocytochemical studies showed marked activation of microglia and astroglia, and cytokine profiling in the cerebral cortex, white matter, and notably in the cerebellum. CSF showed a unique proinflammatory profile of cytokines. These findings indicated that innate neuroimmune reactions play a pathogenic role, at least in some ASD patients. 

Blaylock was the first to explain that excitotoxicity is the central mechanism of F neurotoxicity and the central mechanism in the ASD pathogenesis [40]. Excitotoxicity is caused by excess levels of glutamate and over-activation of ionotropic glutamate receptors on neuronal membranes, leading to ionic influx, disruption of the energy metabolism, and potential neuronal death. In 2008, Blaylock coined the term immunoexcitotoxicity to describe the link between inflammation and excitotoxicity [41]. The complex network of immunoexcitotoxic processes in the pathogenesis of ASD has been explained further in detail [11,42]. Both F and Al^3+^ induce oxidative stress, microglial activation, and the production of inflammatory cytokines and affect neurotransmission. AlF_4_^−^ molecules are triggers of immunoexcitotoxicity and might have a key role in ASD pathogenesis.

### 2.4. Decreased Melatonin as the Potential Marker of ASD

Sleep problems and the early onset of puberty in patients with ASD suggest abnormalities in the melatonin physiology and dysfunctions of the pineal gland. F accumulates in the pineal gland of gerbils treated with fluoridated water for 16 weeks until the time of sexual maturation [16]. Animals excreted less melatonin metabolite in their urine and took a shorter time to reach puberty. The human pineal gland avidly attracts F from the bloodstream because the gland calcifies physiologically with hydroxyapatite. This change takes place even in childhood. F in the apatite crystals averaged about 9000 ppm and in one case went as high as 21,000 ppm in the pineal glands from 11 human corpses [43].

Decreased levels of melatonin in the blood or the urine have been reported as very common features in individuals with ASD compared to typically developing controls. Researchers estimate that 50%–80% of children with ASD suffer from sleep disorders, particularly insomnia [44]. 

The team of twelve French researchers assessed plasma melatonin, whole-blood serotonin, and platelet N-acetyl serotonin in 278 patients with ASD, their 506 first-degree relatives, and 416 sex- and age-matched controls [17]. They confirmed a deficit in melatonin in 51% (45%–57%) as well as hyperserotonemia in 40% (35%–46%) of ASD patients. The melatonin deficit was significantly associated with insomnia. Biochemical impairments were also observed in the first-degree relatives of patients.

**Figure 5:**
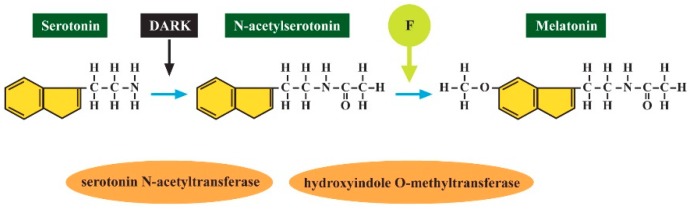
Serotonin is converted to melatonin in dark through the action of two enzymes: serotonin N-acetyltransferase and hydroxyindole O-methyltransferase. F inhibits hydroxyindole O-methyltransferase [47].

The disruption of the serotonin-N-acetyl serotonin-melatonin pathway (Figure 5) was therefore suggested as a biomarker for ASD [17]. To support this hypothesis, another study also investigated the melatonin synthesis in post-mortem pineal glands of 9 ASD patients and 22 controls, in gut samples (11 patients and 13 controls), and blood platelets from 239 ASD patients and 278 controls. The results confirmed the enzymatic mechanism for melatonin deficit in ASD in the pineal gland as well as in the gut and platelets of patients [45]. In a cohort of children drinking water containing 2.5 ppm F, serum serotonin was also increased as compared to controls [46].

Some studies have observed a correlation between abnormal melatonin concentrations and the severity of autistic behaviors [44,48]. Babies with the lowest melatonin production had the most severe neurobehavioral problems. Nocturnal excretion of melatonin was negatively correlated with the problems in the level of verbal language, imitative social play, and repetitive use of objects. 

**Figure 6:**
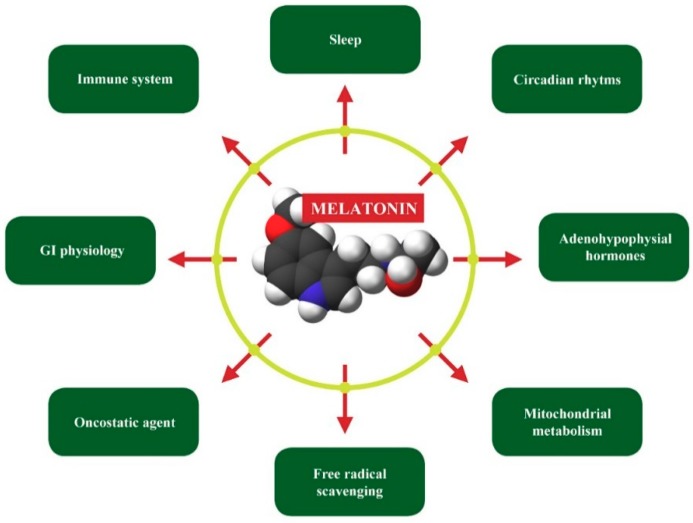
Most important physiological actions of melatonin. Reducing the level of melatonin, F (in yellowgreen) interferes with all indicated events. Image used from Wikimedia Commons according to GNU Free Documentation License.

Melatonin exhibits extraordinary diversity in terms of its functions (Figure 6). 

Melatonin is an important modulator of mitochondrial metabolism, digestive functions, and immunity. It also has a powerful antioxidant effect and increases the levels of several antioxidant enzymes in the brain. F entering the pineal gland and reducing the melatonin synthesis can thus evoke several disruptions of homeostasis, development, and behavior. When combined with the reduced energy production, one can reasonably expect an increase in the vulnerability of neurons and astrocytes to excitotoxicity and oxidative stress.

The recent data for 2065 ASD children aged 4–18 years with sleep disturbance were analyzed to investigate the variation in genes around melatonin synthesis [49]. No significant associations were found between 25 circadian gene variants and sleep problems in this sample of children with ASD. This study, therefore, does not show genetic abnormalities, suggesting that the changes in melatonin synthesis are either secondary to alterations in regulatory pathways or due to F-induced epigenetic changes.

## 3. Fluoride as an Environmental Neurotoxin in the ASD Etiopathogenesis

Research into environmental risk factors for ASD has risen dramatically. According to recent evidence, up to 40%–50% of the variance in ASD liability might be determined by new ecotoxicological factors [6]. How these toxicant exposures may contribute to ASD remains a significant knowledge gap. According to a report by UNICEF in December 1999, fluorosis is endemic in at least 25 countries, such as China and India, Indonesia, South Africa, Iran, and others [50].

F has been linked to neurological and psychiatric disturbances since the 1930s [51]. A sharp decline in mental activity, memory impairment, difficulties with concentration and thinking, and reduced ability to write were observed in aluminum smelter workers and persons living near a factory where F was in high concentrations in the atmosphere [52]. A review of medical evidence in 500 people affected by chronic F intake from artificially fluoridated water appeared in 1977 [53]. The authors made a list of the clinical features, such as chronic fatigue, headaches, loss of mental acuity and the ability to concentrate, depression, a diminished ability to focus, gastrointestinal symptoms and deterioration of muscular coordination. Carlson’s concerns about what increased F levels would do to the developing brain of newborn infants [54] led to the refusal of water fluoridation in most of the European countries. A comprehensive historical review and over 50 papers regarding F neurotoxicity have been provided in the e-book Fluoride Fatigue [51]. 

The surprising observation brought the study of behavior and brain F levels in rats after sodium fluoride (NaF) exposures during late gestation, at weaning, and in adults [55]. Rats exposed prenatally had dispersed behaviors typical of hyperactivity, whereas rats exposed as adults displayed behavior-specific changes typical of cognitive deficits. The accumulations of F were found in all the regions of the brain, with the highest levels in the hippocampus. 

Most of the evidence of F as a developmental neurotoxin in humans has been gathered in China. A study [56] revealed adverse effects of F on the brains of 15 aborted fetuses between five and eight months of gestation from an endemic fluorosis area compared with those from a non-endemic area. This study showed poor differentiation of brain nerve cells and delayed brain development. Purkinje cells of fetuses from the endemic fluorosis area were abnormally disorganized, and a higher nucleus-cytoplasm ratio was observed in brain cones and hippocampus cones. F passing through the placenta of mothers with chronic fluorosis and its accumulation within the brain of the fetus impact the developing central nervous system. A meta-analysis of 16 studies carried out in China between 1998 and 2008 found that children living in an area with a high fluorosis occurrence have five times higher odds of developing a statistically lower IQ than those who live in a low F level area [57].

Choi et al. [58] performed a systematic review and meta-analysis of 27 studies published over 22 years. The authors investigate the effects of increased F exposure and delayed neurobehavioral development of children in China and Iran. Their results revealed the adverse effects of F exposures on children’s neurodevelopment, its potential neurotoxicity, particularly during fetal development and early childhood. These meta-analyses suggest an inverse association between high F exposure and children’s intelligence. From the geographic distribution of the studies, the authors concluded that it seems unlikely that F-attributed neurotoxicity could be attributable to other water contaminants [58]. Based on this study, F was included among the most important developmental neurotoxicants [59]. F differs from most of the current environmental chemicals with impacts on children’s intellectual development in that children are intentionally exposed to it because of its role in the prevention of caries.

The effect of chronic F exposure on children’s intelligence, measured as intelligence quotient (IQ), has been traditionally investigated as an indication of the neurotoxic effect of F in various geographical areas. Over 40 studies published in China, Iran, India, and Mexico found an association between lowered IQ and exposure to F [60,61,62,63,64]. The strength of association between higher F concentrations in the water and children’s reduced intelligence was further supported by a dose-response meta-analysis [65]. These authors evaluated 26 studies of 7258 children and suggested that exposure to F in water should be controlled in areas with high F levels in the water.

The effects of prenatal F exposure and development of children´s cognitive abilities were followed in 299 Mexican mother–children pairs of the Early Life Exposures to Environmental Toxicants birth cohort study [66]. The children’s cognitive ability was evaluated at four years of age using the McCarthy Scales of Children’s Abilities and at 6–12 years of age. Children completed an IQ assessment and provided urine samples for biochemical investigation. Higher levels of F in mothers’ urine during pregnancy were associated with lower cognitive and IQ scores in their children. The authors estimated that each 0.5 mg F/L increase in maternal urinary concentration was associated with an average decrease of 3.15 and 2.50 points in cognitive and IQ scores, respectively. 

The prospective multicenter birth cohort study included 601 mother–child pairs recruited from six major cities in Canada [67]. Children were born between 2008 and 2012, and 41% lived in communities supplied with fluoridated municipal water. Children were between the ages of three and four years at the time of testing IQ scores. Data were analyzed between March 2017 and January 2019. A 1 mg higher daily intake of F among pregnant women was associated with a 3.66 lower IQ score (95% CI, −7.16 to −0.14) in boys and girls. This study found a significant interaction between child sex and maternal urinary F, indicating a differential association between boys and girls. A 1 mg/L increase in maternal urinary F was associated with a 4.49-point lower IQ score (95% CI, −8.38 to −0.60) in boys, but there was no statistically significant association with IQ scores in girls.

We found that 315 laboratory, clinical, epidemiological, and ecological studies over the whole world bring evidence about F neurotoxicity. This is in good agreement with the findings of other authors, who introduced that over 300 animal and human studies indicate that F is neurotoxic [68]. Recently, F has been added to the WHO’s list of *Ten chemicals of greatest public health concern.* Bellinger review brings surveys about how these chemicals adversely affect the brain [69]. To support the case that F may induce neurotoxicity, Bellinger refers to the concern raised by basic neuroscience and ecological studies about the potential effects of excessive F exposure in developing animals and children.

## 4. Is There A Link between ASD Prevalence and a Chronic F Exposure? 

In the past few decades, studies have demonstrated that ASDs occur globally and that the numbers of recorded cases are rising. However, determining the true prevalence figures is still a major challenge. The awareness of ASD, redefinition of diagnostic criteria, the age of investigated children, and the high costs of such surveys have a lot of impact on prevalence figures.

There has been a growing interest in possible environmental factors involved in the etiopathogenesis of ASD. An increasing number of epidemiological reports highlighted the potential link between ASD and chronic F exposure. We attempted to compare the current rates of the ASD prevalence from countries with artificial water fluoridation and the available rates from geographic regions with endemic fluorosis and evaluate the regions with low F supply. While a lot of progress had been made in the global awareness of ASD, much remains to be done to have a more accurate picture of the trend or global burden of the disorder [70]. Our comparison is therefore affected and limited by the available reports. Despite these limitations, the available data are a warning.

The review of Elsabbagh et al. [8] presents the comprehensive tables of available figures of the autism and other pervasive developmental disorders prevalence in various geographic regions over the world in the period of the 1960s–2010. Fifty more studies from 21 countries published during 2000–2016 were analyzed [9], and 27 eligible studies from 18 countries on 5 continents were identified [71]. These reviews demonstrate that the prevalence rates of autism/ASD have increased over time in all investigated regions. 

### 4.1. The ASD Prevalence in Countries with Fluoridated Water

The US is at the top of the list of the current ASD prevalence in developed countries with an average value of 250 per 10,000 (one in 40) children aged 3–17 years [5] (Table 1). It is a further increase since the previous survey in 2014, when one in 59 among eight-year-old children was diagnosed with ASD in the US [72]. CDC reports that 74.4% of the population has been supplied with fluoridated water for 70 years. A large increase in dental fluorosis prevalence was observed in the US [73,74]. In the last survey (2010–2012), dental fluorosis was found in 65 % of adolescents aged 12–15 years. The prevalence rates of ASD are on the rise in Canada. While in 2003, one in 204 children had a diagnosis of autism/ASD, this rate rose to approximately one in 66 in 2018 [75,76]. As of 2007, 45.1% of the Canadian population had access to fluoridated water supplies and water fluoridation remains a contentious issue. The Canadian Health Measures Survey found that 16% of children may have very mild or mild dental fluorosis. Australia, where 80% of the population had access to fluoridated water has revised its ASD prevalence rates from one in 200 in 2012 to an estimated one in 150 people in 2015 [77]. New Zealand reports one person in 66 having ASD [78], and 62% of the total population has fluoridated water supply [79]. 

In countries with fluoridation of public water, it is vital to account additionally for other sources of F intake to assess the public health risk of its chronic exposure. Regarding the potential contribution of F in the ASD etiopathogenesis, one must include that infants and toddlers up to three years receive significantly more F than they should from infant formula. The recommended water F level in the US (0.7–1.2 mg/L) is several times higher than the F level found in the breast milk (0.001–0.004 mg/L) [68]. F intake from other sources including swallowing toothpaste or the use of F tablets in children might further increase the daily F dose.

Tea plants (*Camellia sinensis*) are well known for their ability to accumulate high concentrations of both F and Al^3^ [80]. A cup of black tea could contain 1.4 mg of F. Evidence suggests that the culture of tea drinking in the US, the UK, and New Zealand, contributes significantly to the total body burden of F [80]. In developed countries a high intake of Al^3+^ from food and medicine products is obvious.

### 4.2. The ASD Prevalence in Countries with Endemic Fluorosis

Endemic fluorosis occurs widely in the world and is characterized by skeletal and dental fluorosis and a vast array of bodily pathological changes. Endemic fluorosis has been regarded as a severe public issue in China since the 1960s. There, endemic fluorosis consists of three types: drinking water type, coal-burning type, and drinking brick-tea type. The latter two types only exist extensively in China, but these types are overlapping in some of 34 Chinese provinces [81]. Dental fluorosis occurred in 43%–63% of children aged 8–12 years in endemic areas where the total F intake was 2.7–19.75 mg/day [82].

Recent data show the alarming increase in ASD in China. The last meta-analysis [83] included 44 studies with 2,337,321 children aged 1.6–8 years, covering 30 of the 34 provinces of the country. These studies were conducted between 2000 and 2016. Based on diagnostic criteria, the pooled prevalence of ASD from 16 studies was 39.23 per 10,000, which is lower than in other countries worldwide. However, based on screening tools, the prevalence of ASD ranged from 33 to 1853 per 10,000 with a pooled figure of 429 per 10,000 (one in 23.3). For children in the age group ≤ 4 years, the ASD prevalence was 530 per 10,000 (one in 19) in China (Table 2). Despite the many limitations for the comparison of various ASD prevalence studies that exist, the reports from the last decade revealed that autism prevalence in China is comparable to the Western prevalence [84]. 

A high prevalence of ASD has been reported from Japan (161/10,000) [76]. Recently, the prevalence of ASD in a total population sample of 5-year-old children was estimated at 3.22 % (2.66–3.76) [85]. Dental fluorosis in 18 endemic areas in Japan was described in 1931 by Dean in his classical report [86], which formed the foundation of the concept that the ingestion of F will harden the surface of teeth and make them less susceptible to dental caries. It is interesting that Japan has been practicing a school-based F mouth-rinse program (S-FMR) in nursery schools until graduating from junior high school since 1970 to prevent dental caries [87]. The prevalence of fluorosis is reported as 1.7%–15.4 % of the population in Japan. It seems very probable that children, who use the mouth-rinse every day, swallow F from the mouth-rinse and that this adds to the other F sources from drinking tea, natural waters, and the typical Japanese diet. 

The last available study reports the prevalence of ASD in South Asia ranging from 0.09% in India to 1.07% in Sri Lanka [88]. Preliminary data on the prevalence ASD in a population sample of school children in Eastern India [89] estimate 0.23% (0.07%–0.46%).

An alarmingly high ASD prevalence of 300 per 10,000 (one in 33) was found in Dhaka, the capital city of Bangladesh, which is known to have vast water contamination by various neurotoxicants [88]. In a 2011 survey, South Korea also had a high rate of prevalence (220 per 10,000) [90] (Table 2). 

### 4.3. The ASD Prevalence in the EU Member States

Most of the European states rejected water fluoridation shortly after its introduction in the 1970s–1990s. The Republic of Ireland (Ireland) is the only country in the EU with mandatory artificial fluoridation of drinking water. On the contrary, Northern Ireland, which is the part of the United Kingdom (UK), does not add F to drinking water since the local supply may contain naturally occurring F [91]. In some parts of England, the level of F in the public water supply already reaches 1 mg F/L as a result of the geology of the area. In other areas of the UK, the F concentration has been adjusted by artificial fluoridation. Currently, around six million people in the UK live in areas with fluoridation schemes. Many schemes have been operating there for over 50 years. A high ASD prevalence has been estimated to have been present in the UK since the 1990s (16.8–116 per 10,000) [8,9,71]. The National Autistic Society informs that there are around 700,000 people with ASD in the UK, which is more than one in 100 [92]. 

Unfortunately, there is no central recording of ASD cases in any EU Member State. The European Commission (EC) considered ASD as a rare disease until 2005. The prevalence rates for autism in the EU could be estimated as varying from 3.3 to 16.0 per 10,000 [10,93]. The first pilot project funded by the European Parliament and managed by the EC (ASDEU) provided an estimate of ASD prevalence in 14 European countries in the period 2015–2018 [93]. The program scrutinized 631,619 children aged between seven and nine years. Overall, ASD prevalence estimates varied among European countries from 44 to 197 per 10,000 according to the ASDEU project [93]. However, the estimations from this project are higher than the lower estimates made for the Czech Republic, Poland, Portugal, Finland, France, and Germany [9,71] (Table 3). 

A recent survey of ASD prevalence in the Irish population was performed in 2017 and 2018 as part of an ASDEU project [91,93]. This report indicates an estimated prevalence of autism of 2.9% in school-aged children in Northern Ireland, significantly higher than the 1.5% estimated in the Ireland in the same age group but several years earlier. Waugh et al. showed that the culture of habitual tea drinking in the Ireland could readily exceed the levels known to cause chronic F intoxication in the general population [94], and this is probably relevant to the Irish population in Northern Ireland as well. 

Similarly, an increasing ASD prevalence to 1% was reported from Spain, where fluoridated water has been provided for 10% of the population [9,71]. There are substantial intra-regional differences in ASD prevalence in Sweden, where differences in the F content in drinking water (0.8–1.4 mg F/L) are also well documented [95]. 

### 4.4. The Definition of a Safe Concentration of F for Humans 

An awareness of the potential role of F in the ASD pathogenesis could contribute to the qualified reassessment of its widespread use in the health practice of water fluoridation, which is regarded as a valuable and safe method for reducing dental caries. 

We cannot analyze the history of water fluoridation in the US and the discussion considering F standards. Under the Safe Drinking Water Act, the US Environmental Protection Agency (USEPA) sets the standards for drinking water quality. Currently, the enforceable F standard is set at 4.0 mg/L. The most commonly cited health concerns about F were assessed in the 2006 National Research Council (NRC) report. No evidence substantial enough to support the negative health effects of F at levels below 4.0 mg F/L other than severe dental fluorosis was found [99]. Since 1962, the US Public Health Service (PHS) has recommended an optimal F concentration of 0.7 mg/L and considers that this provides the best balance of protection from dental caries while limiting the risk of dental fluorosis [100]. 

Table 4 provides the latest values for adequate intakes (AI) and upper tolerable intake levels (UL) for F (in mg/day) developed by the National Academies of Sciences, Engineering, and Medicine for the US, updated on 3/27/2019 [101]. The F AI and UL for 0–8-year children were updated in 2017 by the National Health and Medical Research Council (NHMRC) for Australia and New Zealand. This update was intended to prevent dental caries without exceeding intakes that are associated with severe dental fluorosis [102]. Dental fluorosis is caused by overexposure to F during the first eight years of life. The NHMRC did not establish an AI for infants less than six months of age. The review of evidence did not find a reduction in dental caries with F intake in the first six months of life [102].

As F is not an essential nutrient, the panel of the European Food Safety Authority (EFSA) considered that no average requirement for the performance of its essential physiological functions could be defined (12/4/2017) [103]. However, the panel considered that data on the dose–response relationship between caries incidence and the consumption of drinking water with different F concentrations are adequate to set an AI of 0.05 mg F/kg body weight per day for children. This can also be applied to adults, including pregnant and lactating women. However, the AI covers F intake from all sources, including toothpaste and other dental hygiene products. The available data on F intake of the European population is variable but generally at or below 0.05 mg/kg/day [103]. For younger children (1–6 years of age) the UL was exceeded when consuming more than one liter of water at 0.8 mg F and F from other sources. For infants up to six months old receiving infant formula, if the water F level was higher than 0.8 mg/L, the intake of F exceeded 0.1 mg/kg/day, and this level is 100 times higher than the level found in breast milk [104].

The analysis of the literature regarding the loss of IQ after F chronic exposure [68] shows that to protect against a five-point IQ loss, the benchmark dose (BMD) is between 0.0014 and 0.05 mg/day for children. It means that the protective daily dose should be no higher than 0.05 mg/day for children. 

The information now available supports a reasonable conclusion that F exposure of the developing human organism should be minimized. Prolonged exposure to F in the prenatal as well as postnatal stages of development might have neurotoxic effects on the development and metabolism of the brain. Infantile autism develops most likely during the second trimester of prenatal development and results in significant abnormalities in the development of the brain, including the cerebellum, brainstem, and cerebrum. The regressive autism appears to be the result of postnatal events. This form of autism is responsible for the rising ASD incidence in the last decades. Autism-specific brain imaging features were identified at six months of age. Age-specific brain and behavior changes were demonstrated across the first two years of life [105]. This time is a period of intense postnatal synaptic development. The human cerebellum matures postnatally, with the greatest acceleration of growth and neural organization during the first two years after birth [11]. 

However, the dose, at which dental caries reduction is expected, is not far away from the one that might cause chronic pathological effects. It is evident that the definition of a safe concentration of F for humans must consider the fact that in synergy with trace amounts of Al^3+^, every concentration of F might be dangerous for the developing brain. 

### 4.5. Socioeconomic Status

ASD may significantly limit the capacity of some individuals to conduct daily activities. Since it is a life-long condition, it often imposes a significant socioeconomic burden on society, people with these disorders, and their families. In the US, the annual societal costs per the year 2011 for children with ASD were estimated to be between $11.5–60.9 billion, including a variety of direct and indirect costs, from medical care to special education and lost parental productivity [3]. Children and adolescents with ASD had average medical expenditures that exceeded expenditures of those without ASD by $4110–6200 per year.

According to estimates, annual costs due to ASD in the US in 2015 were around $268 billion [106]. This figure is estimated to increase to about $461 billion by the year 2025. If the prevalence of ASD continues to grow as it has in recent years, ASD costs will likely exceed those of diabetes and ADHD by far by 2025 [106]. The economic impact of IQ loss among US children is the loss of tens of billions of dollars [68]. 

The costs of autism for individuals (lifetime) and society (annual) in the UK was calculated in 2005. The average additional lifetime cost for an individual with autism and additional learning disability was estimated to be £2,940,538. For people with high-functioning autism, the additional lifetime cost is estimated to be £784,785 [92].

One of the ASDEU’s key aims was to estimate the economic burden of ASD in the EU. The type of ASD, age, and comorbidities are important drivers of the costs. Estimations of direct costs ranged from €1594 in Romania to €22,378 in Denmark per individual annually. Lifetime costs of caring for a person with autism without an intellectual disability were estimated at €766,865 based on an average life expectancy of 70 years for people with ASD in Germany. The costs of productivity losses among carers range yearly from €615 per carer in Poland to €8934 per carer in Austria [93]. ASDEU also found that the total cost of universal screening of ASD prevalence would range from €43,000 per year in Iceland to €5 million per year in France [93].

These significant health and socioeconomic concerns could probably be lowered by focusing more on ASD prevention through the elimination of F from daily life.

## 5. Discussion

A vast array of observations has been made at the cellular and the molecular levels of the ASD etiopathogenesis. Yet, a unifying mechanism to explain the various etiological factors and different symptoms of ASD has not been proposed. There is a consensus of several researchers that causal processes in the ASD etiopathogenesis involve the interactions of multiple genetic and environmental risk factors. We reviewed literature showing that F is an environmental neurotoxin. F induces symptoms which are observed in people with ASD, such as mitochondrial dysfunction and impairment of energy metabolism, oxidative stress and inflammation, immunoexcitotoxicity, and decreased melatonin levels. We showed that all these symptoms have also been observed in endemic fluorosis areas in China [7], where daily intake of F exceeds the national standard 2 mg per day and might reach up to 20 mg per day [82]. 

Based on the ASD prevalence in various countries and cities, we suggest that F might be the significant culprit in the ASD etiopathogenesis both in areas with artificial water fluoridation as well as in F endemic areas. Nevertheless, F is not yet included among environmental risk factors in the ASD pathogenesis in countries with fluoridated water and a high rate of dental fluorosis. CDC named community water fluoridation one of ten great public health achievements of the 20^th^ century. 

Three main categories of toxicants have been suggested as contributing to the ASD pathogenesis: heavy metals, persistent organic pollutants, and emerging new endocrine disruptors. For example, Modabbernia et al. conducted a review of the 80 studies, nine systematic reviews, and 23 meta-analyses of environmental risk factors for ASD [107]. These authors pointed out that the association between environmental factors and ASD might include gene-related epigenetic effects, oxidative stress, inflammation, endocrine disruption, neurotransmitter alterations, and interference with signaling pathways. Nevertheless, an assessment of F participation in these events was not suggested [107]. 

Ng et al. reviewed 315 articles regarding environmental factors associated with ASD in the years 2003–2013. Research has been conducted worldwide; many studies were concentrated in the US, the UK, Australia, and Japan [108]. However, F was not involved in any of the 315 reviewed articles examining chemical factors [108]. Concerning neurotoxicants, Schofield referred to the current medical databases for 100 molecules or elements that can be listed as developmental neurotoxicants [109]. She reviewed knowledge of the six neurotoxicants, which could be involved in the growing epidemic of neurological illnesses, including autism, over the last 20–25 years. F was not included among the referred neurotoxicants. F did not receive any attention or suspect for participation among environmental factors in the ASD etiopathogenesis, nor in recent reviews of Almandil et al. [6] nor Bjørklund et al. [110]. 

In countries of the EU, where F daily intake is low, F does not probably work as the trigger in the complicated network of pathogenetic events, and ASD is often still considered a rare disease. The document of the EC also concluded that available human studies do not support the conclusion that F in drinking water impairs children’s neurodevelopment at levels permitted in the EU [104].

Many other environmental neurotoxins, exogenous excitotoxic amino acids, and endocrine disruptors can disturb both prenatal as well as postnatal brain development [11,111,112]. Moreover, F from natural waters (e.g., in Sweden and the UK), tea, and non-dietary sources, such as F tablets, toothpaste, and other dental hygiene products, might play a role in the ASD pathogenesis. The severity and the development of ASD symptoms depend on genetics, nutritional status, immune system, and the presence of recurrent infections [11]. Nevertheless, an increase in the ASD prevalence is also observed in some states of the EU with water fluoridation, such as the RoI, the UK, and partially in Spain.

We showed that even very low F concentration in synergy with Al^3+^ can exacerbate alterations in neurotransmission and hormonal regulation. Under such circumstances, a “safe” F concentration might induce pathological effects in children with a genetic susceptibility. The heterogeneity of mutual dynamic interactions can explain the clinically heterogeneous symptoms of ASD and contribute to an understanding of the various responses in any given child to identical environmental neurotoxins.

Anderson [113] suggested in his review that the novel, “emergent” phenomena may arise in individuals with ASD from underlying and interacting genetic and environmental factors. Some of the ASD behavioral symptoms, including delayed stereotyped language, rigid preferences for routines, and repetitive motor mannerisms, among others, can be discussed as potentially being emergent. The rise of ASD in the last decades challenges us to change the research from a reductionistic approach to an understanding of underlying integrative networks. Our review shows how diverse molecules and biological processes can be affected by F.

In the Czech Republic, artificial water fluoridation was stopped in the 1990s for economic reasons, since tap water is used for many purposes. There are many discussions and analyses regarding the efficiency and costs of water fluoridation in the prevention of dental caries over the world. However, the possibility that chronic F intake could evoke chronic diseases with high health and socioeconomic impact would also be involved. The information now available supports a reasonable conclusion that economic losses associated with ASD may be quite large.

Moreover, while ASD is often considered purely behavioral, it comes with many different comorbidities, like gut imbalances, seizures, hormonal disorders, obesity, and sleep problems. Disturbed sleep potentially exacerbates ASD-related symptoms such as impaired social interactions, deficits in nonverbal intelligence and communication, repetitive behaviors, and mood disorders. Poor sleep may have adverse effects on children’s attention, memory, learning, and conduct aggression.

The ASD prevalence is consistently estimated as a ratio of approximately 4.5 male:1 female during 2006–2014 in the US [3,70]. For example, the pooled ASD prevalence was one in 38 boys and one in 152 girls. However, the male-to-female prevalence ratios ranged from 2.7 in Utah to 7.2 in Alabama [72]. The perspective that ASD could impact around 30% of young men in the next decades is very alarming and requires urgent solutions. The reduction of F and Al^3+^ consumption of pregnant women and developing children could be a very easy and inexpensive way to prevent ASD. 

## 6. Conclusions

The rise in the ASD prevalence in countries with water fluoridation as well as in endemic fluorosis areas supports a view that F is an important environmental factor in the ASD etiopathogenesis. Our suggestion of the important role of F in the ASD etiopathogenesis is supported by the observation that a high ASD rate is found in countries with a high occurrence of dental fluorosis.

F neurotoxicity has been demonstrated in many laboratory studies with cells and animals, as well as in human epidemiological studies. We present evidence that F induces mitochondrial dysfunction, oxidative stress, inflammation, and immunoexcitotoxicity. There is a link between a known effect of F on melatonin synthesis in the pineal gland and the finding that melatonin synthesis is significantly reduced in ASD. Moreover, understanding F-induced pathways in the ASD etiopathology may lead to novel treatments. All these F-induced symptoms could evoke several disruptions of the brain development, alter neurotransmission and hormonal regulations, deficits in social interactions and induce repetitive, stereotypic interests, and behaviors resulting in ASD. 

At present, there is a divergence between public health practice of water fluoridation, which is regarded as valuable and safe for reducing dental caries, and current scientific evidence, which indicates that F is a neurotoxin disturbing prenatal as well as postnatal brain development, eroding intelligence, and behavior. The potential neurotoxicity associated with exposure to F, which has generated controversy about community water fluoridation, remains unclear. In the recent Canadian study, maternal exposure to higher levels of F during pregnancy was associated with lower IQ scores in children aged 3 to 4 years [67]. These findings support the possible need to reduce F intake during pregnancy. Intellectual disability is present in 65%–75% of individuals with a diagnosis of autistic disorder and in 30%–55% of all ASD [3].

F is not an essential nutrient as no physiological function can be defined for which F is required. The presence of trace amounts of Al^3+^ strongly potentiates the neurotoxic effects of F. AlF_4_^−^ can trigger the pathological symptoms of ASD at concentrations several times lower than those for F acting alone. In synergy with Al^3+^ every concentration of F might be dangerous for the developing brain. Our review suggests that the reduction of F exposure in daily life might be an efficient way to prevent an ASD epidemic soon. Monitoring of the ASD prevalence in children born after the removal of F from drinking water will provide relevant information for our hypothesis. 

## Figures and Tables

**Table 1:** Current ASD prevalence in countries with water fluoridation.

Country Year	Prevalence per 10,000	This is 1 in X Children	Water Fluoridation% of Population	Reference
US 2014	169	1:59	70% for 70 y	[72]
US 2016	250	1:40	70% for 70 y	[5]
Canada 2018	152	1:66	45% for 12 y	[75,76]
New Zealand 2016	152	1:66	62% for 50 y	[78,79]
Australia 2015	144	1:150	80% for 35 y	[77]

**Table 2:** ASD prevalence in countries with endemic fluorosis.

Country Year	Prevalence per 10,000	This is 1 in X Children	Reference
Bangladesh 2016	15; 80	1:666; 1:125	[88]
Dhaka 2016	300	1:33	[88]
China 2013–2016	19; 42	1:526; 1:238	[9,83]
China 2016	429; 530	1:23; 1:19	[83]
China 2013 Jilin	108	1:92.5	[84]
Japan	161	1:62	[76]
Japan 2018	322	1:31	[85]
India 2016	9	1:1111	[88]
India 2017	23	1:435	[89]
South Korea 2011	220	1:45	[90]
Sri Lanka 2016	93	1:107	[88]

**Table 3:** The ASD prevalence in the EU member states according to the available last reports.

Country	Prevalence per 10,000	This is 1 in X Children	Reference
EU total 2015–2018	44–197, average 122	1:82	[93]
Belgium	60	1:167	[96]
Czech Republic	12	1:833	(pers. comm.)
Denmark	34; 68	1:294; 1:147	[76,96]
Finland	77	1:130	[91]
France	27; 36	1:370; 1:277	[97]
Germany	38	1:263	[96]
Ireland	150	1:66	[91]
Italy Pisa	86	1:116	[91,98]
Netherland	57, 84	1:175; 1:119	[9,71]
Northern Ireland	290	1:35	[91]
Norway	12; 70	1:833; 1:142	[9,91,96]
Poland	3	1:3333	[96]
Portugal	9.2	1:1086	[9,71,76]
Spain	13; 100	1:769; 1:100	[9,71,76]
Sweden	71; 115	1:141; 1:87	[9,71,91]
UK	100	1:100	[92]

**Table 4:** Adequate intake (AI) and upper tolerable intake levels (UL) for F (in mg/day). According to the National Academies of Sciences, Engineering, and Medicine, US [101]; NHMRC, Australia and New Zealand [102]; and EFSA, EU [103].

Age	US		Australia, NZ		EU	
	AI	UL	AI	UL	Age	AI
0–6 m	0.01	0.7	–	1.2	0–6 m	–
7–12 m	0.5	0.9	0.5	1.8	7–11 m	0.4
1–3 y	0.7	1.3	0.6	2.4	1–3 y	0.6
4–8 y	1.0	2.2	1.1	4.4	4–6 y	1.0
9–13 y	2.0	10	2.0 *; 3.0 ^+^	10	7–10 y	1.5
14–18 y	3.0	10	2.0 *; 3.0 ^+^	10	11–14 y	2.2
Males	4.0	10	4.0	10	15–17 y	3.2
Adult females	3.0	10	3.0	10	Adults	3.4

The following reference body weights were used when the AI and UL were expressed in mg F/day: 0–6 months, 6 kg; 7–12 months, 9 kg; 1–3 years, 12 kg; 4–8 years, 22 kg; 9–13 years, 40 kg; boys aged 14–18 years, 64 kg; 14–18 year-old girls, 57 kg; 76 kg for adult men; and 61 kg for adult women. * girls, ^+^ boys.
